# North American Propolis Extracts From Upstate New York Decrease *Nosema ceranae* (*Microsporidia*) Spore Levels in Honey Bees (*Apis mellifera*)

**DOI:** 10.3389/fmicb.2020.01719

**Published:** 2020-07-22

**Authors:** Andre J. Burnham, Emily De Jong, Jayre A. Jones, Herman K. Lehman

**Affiliations:** ^1^Department of Biology, Hamilton College, Clinton, NY, United States; ^2^Program in Biochemistry, Hamilton College, Clinton, NY, United States; ^3^Department of Pediatrics, Emory University School of Medicine, Atlanta, GA, United States; ^4^Program in Neuroscience, Hamilton College, Clinton, NY, United States

**Keywords:** *Nosema ceranae*, microsporidia, nosemosis, propolis, fumagillin, dichloromethane, natural product extract

## Abstract

*Nosema ceranae* infections in honey bees (*Apis mellifera*) pose a severe threat to colony health. Beekeepers have used dicyclohexylammonium fumagillin to control *Nosema apis*, although it may be ineffective against *N. ceranae*. We investigated the ability of various propolis extracts collected from Upstate New York (United States) to decrease *in vivo N. ceranae* infection levels when fed *ad libitum* to *N. ceranae*-infected honey bees. Propolis extracts, most notably a dichloromethane extract, significantly lowered spore levels in a dose-dependent fashion 4 days post inoculation. When testing the *in vitro* anti-*Nosema* activity of propolis extracts, we report for the first time that spore viability was unaffected after a 24 h exposure to propolis extracts. These results present evidence that propolis extracts may effectively lower *Microsporidia* infections in honey bees, and that direct exposure of environmental spores to propolis alone does not kill *N. ceranae*.

## Introduction

Nosemosis is a prevalent bee disease caused by fungal microsporidian parasites, *Nosema apis* and *Nosema ceranae* (Chen and Huang, 2010). Nosemosis type A is caused by *N. apis* while nosemosis type C is caused by *N. ceranae* (Higes et al., 2010). *N. ceranae* is now considered the more virulent of the two species and has recently developed a wide geographical distribution, including in North America (Klee et al., 2007; Chen et al., 2008; Higes et al., 2010). *N. ceranae* has been linked to a range of actions on honey bees (*Apis mellifera*) including immunosuppression, lipid loss, and impairment of foraging and homing behavior, honey production, brood rearing, and bee and colony survival (Antúnez et al., 2009; Higes et al., 2009, 2013; Botías et al., 2013; Li et al., 2018).

*Nosema ceranae* is a spore-forming obligate intracellular parasite whose reproductive cycle initiates in the honey bee midgut lumen after being orally ingested by an adult bee (Higes et al., 2007; Texier et al., 2010; Smith, 2012). Mature environmental spores germinate in the host digestive lumen, and a polar filament (a microsporidian invasion organelle) injects infectious sporoplasm into host epithelial cells (Texier et al., 2010; Gisder et al., 2011). Sporoplasm forms primary meronts, which then proliferate (merogony) and mature into primary spores which may autoinfect the same cell or adjacent cells (Franzen, 2004; Han and Weiss, 2017). Secondary meronts are then formed and may be released back into the lumen by cell lysis as environmental spores or excreted in host feces and infect other animals (Fries and Granados, 1992; Franzen, 2004; Gisder et al., 2011; Smith, 2012; Han and Weiss, 2017). The vegetative cycle is complete 4 days post infection, and high numbers of primary and environmental spores are found in the host within this time period (Gisder et al., 2011).

Bicyclohexylammonium fumagillin (fumagillin) is an antimicrobial agent originally isolated from the fungus, *Aspergillus fumigatus*, and has been an effective treatment for *N. apis* infections for more than 50 years (Bailey, 1953; van den Heever et al., 2014). In contrast, fumagillin may not be as effective against *N. ceranae* infections (Kochansky and Nasr, 2004; Pajuelo et al., 2008; Williams et al., 2008; Higes et al., 2011; Huang et al., 2013; Giacobino et al., 2016; van den Heever et al., 2016; Mendoza et al., 2017). Furthermore, the toxicity associated with fumagillin has resulted in tight regulations on its use in many countries (van den Heever et al., 2014). Thus, alternative therapies for nosemosis in honey bees are needed.

Honey bee propolis, which is composed of tree resin, pollen, nectar, bees wax, and other organic materials, is produced by bees and used as cement to seal hive cracks and crevasses (Huang et al., 2014). Interestingly, it has also been postulated that honey bee propolis may have colony self-medication effects against various parasites (Simone-Finstrom et al., 2009; Simone-Finstrom and Spivak, 2010, 2012; Erler and Moritz, 2016), although it was recently reported that bees do not use propolis to self-medicate against *Nosema* infections (Mura et al., 2020).

In addition to self-medication effects, propolis components (e.g., ethanolic extracts) harvested from bees in Asia and Europe have been shown to lower mortality and *Nosema* infections in Asian honey bees (*Apis cerana*), dwarf honey bees, (*Apis florea*) and European honey bees (Yemor et al., 2015; Suwannapong et al., 2018; Mura et al., 2020). Recently, Arismendi et al. (2018) reported that methanolic Chilean propolis extracts reduce *N. ceranae* loads and increase survival of European honey bees (*A. mellifera*). Mura et al. (2020) reported that ethanolic Spanish propolis extracts reduce *N. ceranae* loads in European honey bees and identified high concentrations of caffeic acid, ferulic acid, ellagic acid, and quercetin derivatives in their extracts. Bee species, extraction methods, and especially geographical origin of propolis all have an effect on its bioactivity, most likely on account of variations in chemical composition (Huang et al., 2014). The efficacy of propolis components originating in North America, and the potency and dosage of different solvent extracts as *N. ceranae* treatments remain underexplored. In addition, the *in vitro* activity of propolis extracts on *N. ceranae* spore viability has yet to be studied. Here, we have tested the effect of North American propolis extracts, prepared in various solvents and concentrations, on *N. ceranae* infection levels in European honey bees, and we have investigated the *in vitro* activity of these extracts on spore viability.

## Materials and Methods

### Spore Purification

*Nosema ceranae* spores were purified from infected local honey bees (Location: 43.050215, −73.414246), adapted from Green et al. (2000). Approximately 80 bee abdomens were dissected, homogenized in 8 ml distilled water, filtered once through 2 mm mesh and twice through a 70 μm mesh sieve. Homogenates were centrifuged for 5 min at 5000 × *g* at 4°C. The resulting spore pellet was overlaid on a 50% Percoll, 50% tris-buffered saline (TBS) solution and centrifuged for 30 min at 500 × *g* at 4°C. The purified spore pellet was then washed and re-pelleted in 1× TBS. In order to initiate infection within the honey bee digestive tract (see below), spore pellets were air dried and resuspended (4 × 10^7^ cells/ml) in 5% sucrose solution (Olsen et al., 1986). Thereafter, *N. ceranae* were identified with conventional PCR methods. Extracted DNA (20 μg) from ca. 10^6^ spores, oligonucleotide primers (218MITOC-FOR, 218MITOC-REV for *N. ceranae* and 321APIS-FOR and 321APIS-REV for *N. apis*), and Taq polymerase were combined and PCR conditions were completed, as described by Martín-Hernández et al. (2007). Of 10 samples collected from a heavily infected colony, all tested negative for *N. apis* and positive for *N. ceranae* (Supplementary Figure S1). Purified spores from this colony were used in all experiments described below.

### Preparation of Propolis Extracts

The molecular composition of propolis is known to vary greatly depending on factors related to geographical origin (e.g., plant sources); our propolis was collected from New York, United States, and we herein define propolis and propolis extracts as Upstate New York propolis (UNYP). UNYP extracts were prepared from raw propolis collected from the New York state apiary (43.100108, −73.510797). UNYP, (10 g) was dried for 48 h (45°C) and extracted in 100 ml of 70% ethanol. Batches of ethanol extracts were dried by roto-evaporation, and 10 g aliquots were extracted with 100 ml of a 9:1 methanol/water solution and then extracted twice with 50 ml hexanes. After removing the hexane layer, the remaining methanolic layer was diluted with 50 ml of water and extracted twice with 40 ml dichloromethane (DCM). Ethanol, methanol, and DCM extract fractions were separately dried and re-dissolved in 70% ethanol. Maximum miscible concentrations were determined by the mass of extract that could be dissolved without precipitation in a 7% ethanol vehicle solution in 50% sucrose: ethanol-extracted UNYP, 5.9 g/l; methanol /water-extracted UNYP, 13.8 g/l; DCM-extracted UNYP, 2.6 g/l.

### Animal Inoculation Experiments

The effect of UNYP extracts on *N. ceranae* infection levels was tested by infecting honey bees with fixed concentrations of microsporidia and feeding UNYP extracts to individual honey bees. Sealed frames of brood were collected from local, *Nosema*-free colonies and placed in a growth chamber (34°C and 44% humidity). Emerging bees were separated from the growth chamber 24 h after hatching and placed into individual cages for a 4-h starvation period, prior to being inoculated with a 5 μl suspension containing 2 × 10^5^ *N. ceranae* spores (see above). Bees in individual cages were returned to the growth chamber (34°C and 44% humidity) and feeder-fed either a positive control (50% sucrose), vehicle control (7% ethanol in 50% sucrose), ethanol extract (in 50% sucrose plus vehicle), methanol extract (in 50% sucrose plus vehicle), or DCM extract (in 50% sucrose plus vehicle), for 4 days *ad libitum.* Negative controls were not inoculated with spores and fed only sucrose solution. There was no significant difference in the amounts consumed per bee between treatments (34 ± 2 μl; Supplementary Figure S2). Four days post inoculation (dpi), the number of *N. ceranae* spores were isolated from individual bee abdomens (*n* = 23–44, biological replicates) from each treatment group, suspended in distilled water, and quantified with a hemocytometer (Cantwell, 1970).

### *In vitro* Spore Viability Assays

To test if UNYP extracts were responsible for directly killing *N. ceranae* spores, we used an *in vitro* spore viability assay modified from Ptaszyńska et al. (2018). Following purification, 10^7^ spores were incubated at room temperature in 1 ml of UNYP extracts (2.6 g/l) or a 7% ethanol vehicle control in 5% sucrose for 24 h. Heat killed control spores were incubated at 95°C for 1 min (Green et al., 2000). Following each treatment, we stained spores with SYTOX Green (Molecular Probes, Inc., Eugene, Oregon) and visualized samples with fluorescent confocal microscopy (504/523 nm) to view dead or inviable microsporidia (Green et al., 2000). The percentage of inviable spores was quantified by counting the total number of live (unlabeled) and dead (labeled) spores from two images per sample. Three replicated *in vitro* experiments were performed.

### Statistical Analyses

Statistics and p values were generated using one-way and two-way ANOVAs and Tukey’s multiple comparisons tests in Prism version 6.0c.

## Results and Discussion

**FIGURE 1:**
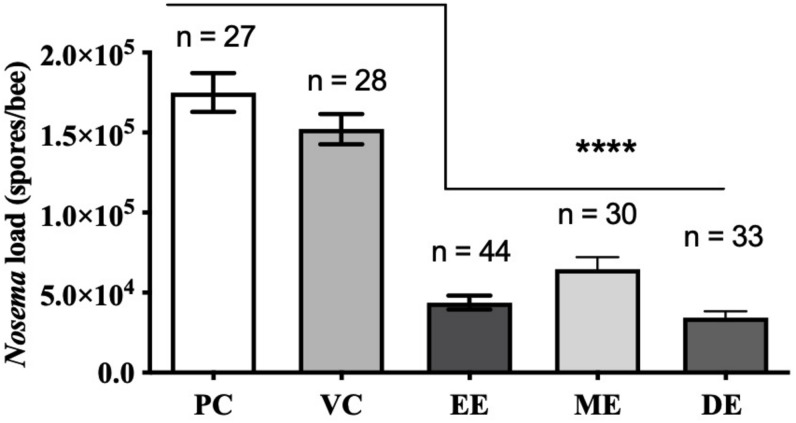
Honey bee *N. ceranae* spore levels 4 days post inoculation (dpi; 2 × 10^5^ spores) following feeding infected worker bees with maximum soluble concentrations of Upstate New York propolis (UNYP) extracts. Individual animals were fed *ad libitum* ethanol extract (EE; 5.9 g/l), methanol/water extract (ME; 13.8 g/l), DCM extract (DE; 2.6 g/l), positive control (PC), or vehicle control (VC). Each bar represents the mean ± SEM and the number of bees in each treatment group is indicated (****indicates *p* ≤ 0.0001).

**FIGURE 2:**
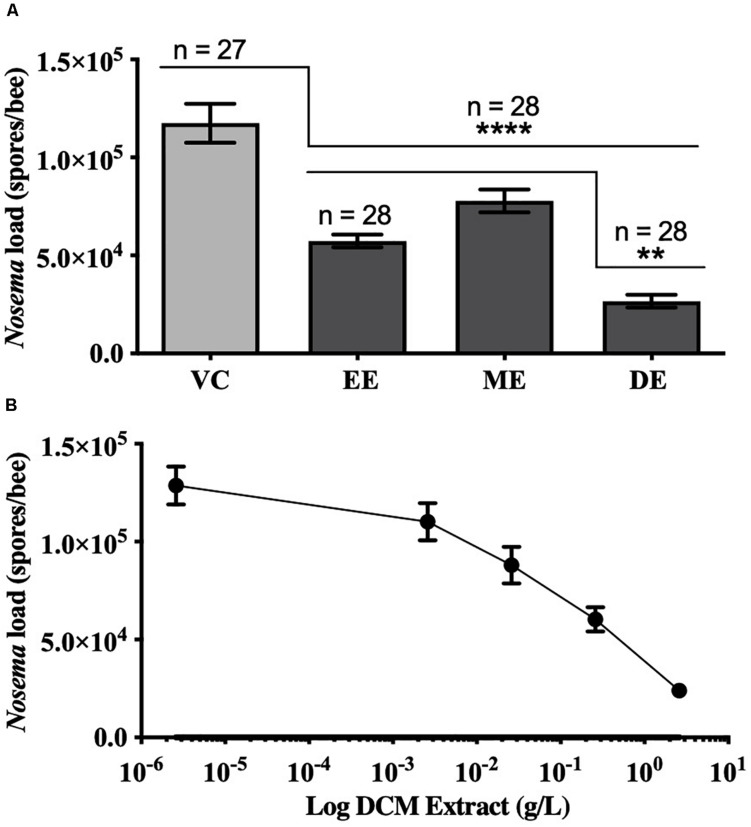
Honey bee *N. ceranae* spore levels 4 days post inoculation (dpi; 2 × 10^5^ spores) following feeding infected worker bees with similar concentrations of different UNYP extracts. **(A)** Individual bees were fed *ad libitum* ethanol extract (EE, 2.6 g/l), methanol extract (ME, 2.6 g/l), dichloromethane extract (DE, 2.6 g/l), or a vehicle control (VC). All values represent the mean spore load ± SEM and the number of bees in each treatment group are indicated (**indicates *p* ≤ 0.01 and ****indicates *p* ≤ 0.0001). **(B)** Individual bees were fed *ad libitum* with different concentrations of dichloromethane (DCM) extracts (10-fold dilutions of 2.6 g/l DCM extract). All values represent the mean spore load per bee ± SEM (*n* = 23 bees per dose).

**FIGURE 3:**
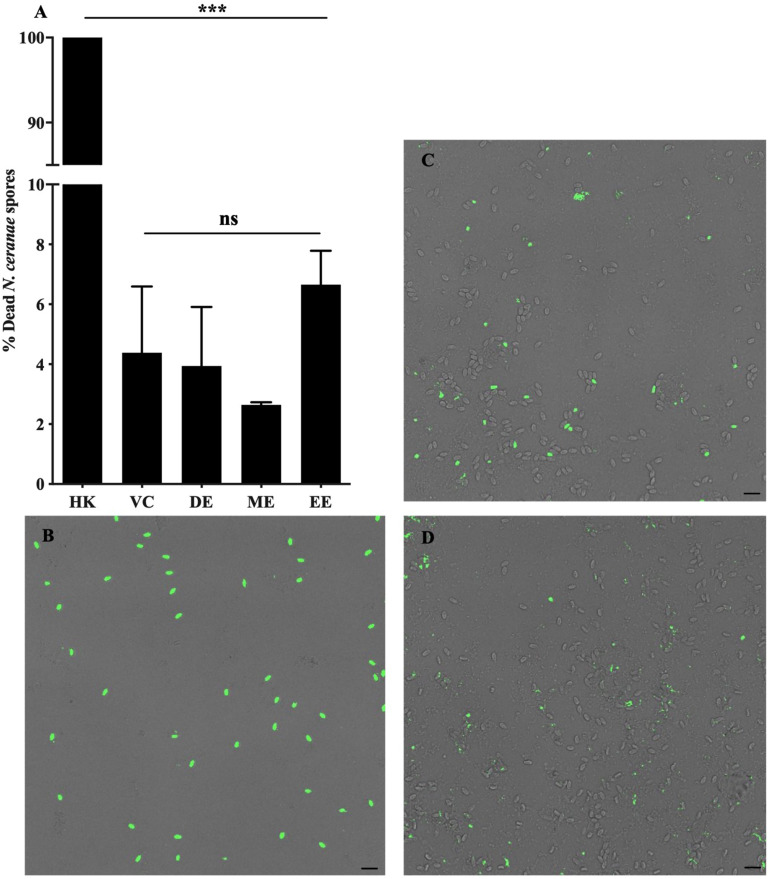
*Nosema ceranae* spore viability following treatment with UNYP extracts *in vitro*. **(A)** Purified living spores were treated with dichloromethane extract (2.6 g/l, DE), methanol/water extract (2.6 g/l, ME), ethanol extract (2.6 g/l, EE), or a 7% ethanol vehicle control (VC) in 5% a sucrose solution for 24 h. Heat-killed spores (HK) served as a cell death control. Spores were fluorescently labeled with a nuclear cell viability dye following treatment and visualized with confocal fluorescent microscopy. Each value represents the mean ± SEM (*n* = 3, ***indicates *p* ≤ 0.001). Representative fluorescent and bright field overlaid images of **(B)** heat killed spores (HK), **(C)** spores treated with VC, and **(D)** spores treated with DE are shown. Scale bar = 10 μm.

Honey bees fed UNYP extracts had significantly lower *N. ceranae* spore levels when compared to positive and vehicle controls. Spore levels significantly decreased when animals were treated with maximum miscible concentrations of ethanol [*F*(2,96) = 83.17, *p* < 0.0001], methanol [*F*(2,82) = 36.55, *p* < 0.0001], and DCM extracts [*F*(2,84) = 77.04, *p* < 0.0001; Figure 1]. UNYP extracts tested at the same concentrations (2.6 g/l for ethanol, methanol, and DCM extracts) also resulted in lower spore counts as compared to positive and vehicle controls [*F*(3,88) = 37.69, *p* < 0.0001; Figure 2A]. The DCM extract exhibited the greatest reduction in spore levels followed by ethanol and methanol/water extracts (*p* = 0.004; Figure 2A). Moreover, DCM extracts displayed a dose-dependent effect; as DCM concentrations increased, spore levels decreased [*F*(4,109) = 26.76, *p* < 0.0001; Figure 2B]. Negative control animals maintained spore levels of zero in all inoculation experiments. In our *in vitro* assays, we found no effect of UNYP extracts on spore viability (Figures 3A–D).

Given that the *N. ceranae* vegetative cycle is complete by day 4 of infection (Gisder et al., 2011), we chose to sacrifice animals 4 dpi in our cage trials. Others have previously shown that propolis extracts significantly decrease honey bee mortality and *N. ceranae* spore counts with longer infection periods (Yemor et al., 2015; Arismendi et al., 2018; Suwannapong et al., 2018). However, we did not measure honey bee survival in this study, as we were focused on the effects of various UNYP extracts and concentrations on spore load *in vivo* and *in vitro*.

We observed that UNYP lowered *N. ceranae* infections, but it is unknown which compounds are responsible for this activity. Many anti-infective molecules (e.g., organic acids and flavonoids) have been isolated from propolis (Marcucci, 1994; Toreti et al., 2013; Huang et al., 2014; Mura et al., 2020). Flavonoids, terpenoids, phenolics, and a variety of other aromatic molecules are the main chemical constituents of propolis (Toreti et al., 2013). More specifically, caffeic acid (a phenolic acid) and pinocembrin (a flavonoid) have both been found to inhibit fungal growth (Toreti et al., 2013). Microsporidia are fungi, and it is possible, therefore, that the anti-*Nosema* activity contained within our extracts is attributable to phenolic acids, flavonoids, or other antifungal compounds common to propolis. However, a synergistic interaction of a variety of compounds may also be responsible for our observations.

The DCM extract likely contained the most potent compound(s) since it demonstrated the greatest effect of treatment. These results agree with previous studies finding DCM propolis extracts to have the highest antifungal activity relative to more polar organic or aqueous fractions (Johann et al., 2007; Boisard et al., 2015; Afrouzan et al., 2017). Interestingly, pinocembrin has been detected in DCM propolis extracts (Boisard et al., 2015; Afrouzan et al., 2017). An in-depth characterization of our UNYP extracts and bioassays is needed to identify specific compounds that could be responsible for our results, especially since the chemical composition of propolis may vary between regions, and the molecular composition of propolis from North American regions has not been well studied (Huang et al., 2014).

Our *in vitro* data are of particular interest. *N. ceranae* spore viability was unaffected by direct UNYP treatment *in vitro*. This finding suggests that the honey bee internal environment is likely necessary for the anti-*Nosema* activity of propolis and we hypothesize that the UNYP agents examined in this study require metabolic modification, impair the microsporidian reproductive cycle, or perhaps modulate immune function. For instance, previous works have suggested that propolis extract ingestion may modulate expression of various bee immune and metabolic genes, including *defensin1, abaecin, hymenoptaecin*, and *P450s* (Simone-Finstrom et al., 2009, 2017; Johnson et al., 2012). Further investigations focused on the mechanism of action of UNYP and other propolis extracts would be beneficial in order to better understand its applications to apicultural medicine.

## Conclusion

Herein, we demonstrated for the first time that oral treatments of North American propolis (UNYP) extracts, most notably a DCM extract, significantly lowered *N. ceranae* spore levels in European honey bees. We further report that DCM UNYP extracts have dose-dependent activity and significantly higher potency than ethanol and methanol extracts on *N. ceranae* infections. We are also the first to show that *in vitro N. ceranae* spore viability is unaffected by propolis extracts. Due to the vast array of plant species native to different regions of North America, the biological activity our UNYP extracts is not necessarily representative of propolis harvested from other regions of North America. Nevertheless, these data provide insight for future investigations focused on responsible molecules and the anti-microsporidian mechanisms of action of UNYP and propolis from other regions. Considering that fumagillin is considered environmentally toxic and its efficacy on targeting *N. ceranae* is questionable, components of propolis extracts may present a natural alternative treatment. However, since bees are not known to consume propolis in nature, continued toxicity testing, chemical composition analyses, and larger colony-wide trials are needed. Our results have important implications toward improving veterinary and apicultural methods, and provide evidence that propolis may be used to treat *N. ceranae* in honey bees and perhaps be applied to future microsporidiosis research in alternative medicine.

## Data Availability Statement

All datasets presented in this study are included in the article/Supplementary Material.

## Author Contributions

AB conceived the project, designed the study, participated in all aspects of the acquisition, analysis and interpretation of the data, wrote drafts and revisions of the manuscript, approved the final version of the manuscript, and was accountable for all aspects of the work. ED participated in the acquisition, analysis and interpretation of the animal inoculation experiments, and approved the final version of the manuscript. JJ participated in the data analysis and interpretation, and approved the final version of the manuscript. HL assisted with the project concept, and participated in all aspects of the study design, acquisition, analysis and interpretation of the data, wrote drafts and revisions of the manuscript, wrote the final version of the manuscript and was accountable for all aspects of the work. All authors contributed to the article and approved the submitted version.

## Conflict of Interest

The authors declare that the research was conducted in the absence of any commercial or financial relationships that could be construed as a potential conflict of interest.

## Supplementary Material

FIGURE S1 Identification of *Nosema ceranae* spores. **(A)** Microscopic view of purified *N. ceranae* spores used in this study. **(B)** Gel electrophoresis of PCR products from spore samples using primers for *N. ceranae*, *N. apis*, and β-actin. Samples 1 and 2 were collected at the beginning and end of this study, respectively. Microscopy and PCR results indicate that spore preparations consist of *N. ceranae*.

FIGURE S2 Treatment solutions consumed by bees in 24 h. Individual bees were housed in feeding chambers with feeders containing treatment solutions attached to chambers. Feeders were weighed before and after 24-h feeding periods. After controlling for evaporation, final weights were subtracted from initial weights in order to calculate the mass of food consumer per 24 h. Volumes consumed per bee were then determined according to the calculated density of known sample volumes and weights. All treatments were stained with blue food dye and animals dissected to confirm that bees had ingested treatment solutions. Each bar represents the mean ± SEM (n.s., not statistically different; *n* = 5).
