# Supplementary figures and images for: North American Propolis Extracts From Upstate New York Decrease Nosema ceranae (Microsporidia) Spore Levels in Honey Bees (Apis mellifera)
Source: Front Microbiol. 2020 Jul 22;11:1719. doi: 10.3389/fmicb.2020.01719 (PMC7387503; doi:10.3389/fmicb.2020.01719)

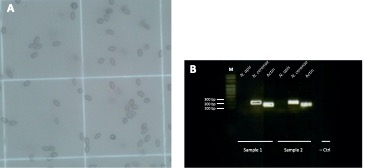

Supplement: FIGURE S1 — Identification of Nosema ceranae spores. (A) Microscopic view of purified N. ceranae spores used in this study. (B) Gel electrophoresis of PCR products from spore samples using primers for N. ceranae, N. apis, and β-actin. Samples 1 and 2 were collected at the beginning and end of this study, respectively. Microscopy and PCR results indicate that spore preparations consist of N. ceranae. [file Image_1.jpeg]

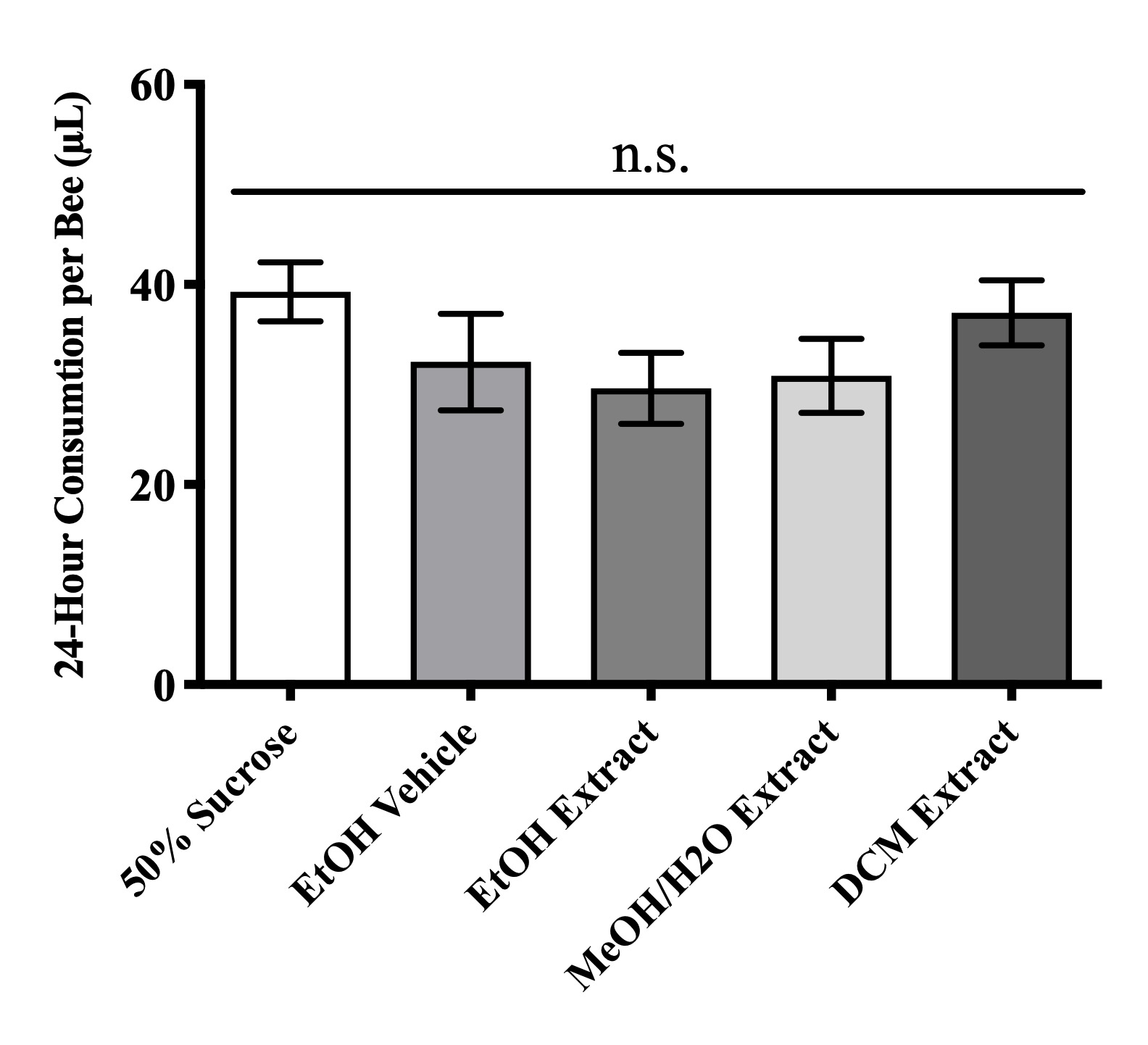

Supplement: FIGURE S2 — Treatment solutions consumed by bees in 24 h. Individual bees were housed in feeding chambers with feeders containing treatment solutions attached to chambers. Feeders were weighed before and after 24-h feeding periods. After controlling for evaporation, final weights were subtracted from initial weights in order to calculate the mass of food consumer per 24 h. Volumes consumed per bee were then determined according to the calculated density of known sample volumes and weights. All treatments were stained with blue food dye and animals dissected to confirm that bees had ingested treatment solutions. Each bar represents the mean ± SEM (n.s., not statistically different; n = 5). [file Image_2.jpeg]
